# Radiation upregulates macrophage TREM-1 expression to exacerbate injury in mice

**DOI:** 10.3389/fimmu.2023.1151250

**Published:** 2023-04-24

**Authors:** Satoshi Yamaga, Atsushi Murao, Gaifeng Ma, Max Brenner, Monowar Aziz, Ping Wang

**Affiliations:** ^1^ Center for Immunology and Inflammation, The Feinstein Institutes for Medical Research, Manhasset, NY, United States; ^2^ Departments of Surgery and Molecular Medicine, Zucker School of Medicine at Hofstra/Northwell, Manhasset, NY, United States

**Keywords:** TREM-1, macrophage, ionizing radiation, eCIRP, DAMP

## Abstract

**Introduction:**

Exposure to high-dose ionizing radiation causes tissue injury, infections and even death due to immune dysfunction. The triggering receptor expressed on myeloid cells-1 (TREM-1) has been demonstrated to critically amplify and dysregulate immune responses. However, the role of TREM-1 in radiation injury remains unknown. Extracellular cold-inducible RNA-binding protein (eCIRP), a new damage-associated molecular pattern, is released from activated or stressed cells during inflammation. We hypothesized that ionizing radiation upregulates TREM-1 expression via eCIRP release to worsen survival

**Methods:**

RAW264.7 cells and peritoneal macrophages collected from C57BL/6 wild-type (WT) mice were exposed to 5- and 10-Gray (Gy) radiation. C57BL/6 WT and CIRP-/- mice underwent 10-Gy total body irradiation (TBI). TREM-1 expression on RAW264.7 cells and peritoneal macrophages in vitro and in vivo were evaluated by flow cytometry. eCIRP levels in cell culture supernatants and in peritoneal lavage isolated from irradiated mice were evaluated by Western blotting. We also evaluated 30-day survival in C57BL/6 WT, CIRP-/- and TREM-1-/- mice after 6.5-Gy TBI.

**Results:**

The surface protein and mRNA levels of TREM-1 in RAW264.7 cells were significantly increased at 24 h after 5- and 10-Gy radiation exposure. TREM-1 expression on peritoneal macrophages was significantly increased after radiation exposure in vitro and in vivo. eCIRP levels were significantly increased after radiation exposure in cell culture supernatants of peritoneal macrophages in vitro and in peritoneal lavage in vivo. Moreover, CIRP-/- mice exhibited increased survival after 6.5-Gy TBI compared to WT mice. Interestingly, TREM-1 expression on peritoneal macrophages in CIRP-/- mice was significantly decreased compared to that in WT mice at 24 h after 10-Gy TBI. Furthermore, 30-day survival in TREM-1-/- mice was significantly increased to 64% compared to 20% in WT mice after 6.5-Gy TBI.

**Conclusion:**

Our data indicate that ionizing radiation increases TREM-1 expression in macrophages via the release of eCIRP, and TREM-1 contributes to worse survival after total body irradiation. Thus, targeting TREM-1 could have the potential to be developed as a novel medical countermeasure for radiation injury.

## Introduction

High doses of ionizing radiation exposure, which can be caused by terrorism, nuclear warfare, and accidents at nuclear power plants lead to severe infections and death due to hematopoietic and gastrointestinal syndrome accompanied by immune cell depletion and bacterial translocation (1–4). Preserving the normal immune function combats infection and is one of the pivotal elements of the management of radiation injury. Neutrophils and macrophages are important cell types in innate immunity. The number of circulating neutrophils is markedly reduced by high doses of ionizing radiation due to their short half-life in peripheral blood and the radiosensitivity of bone marrow (5). This would suggest that macrophages, which are more radioresistant, may play a crucial role in host defense against infections after radiation injury.

The triggering receptor expressed on myeloid cells-1 (TREM-1) can be expressed on monocytes, macrophages, neutrophils, and dendritic cells. TREM-1 has been identified as a critical amplifier of inflammation to dysregulate immune response in various diseases (6). In septic patients, TREM-1 is upregulated on monocytes (7) and neutrophils (8). A soluble form of TREM-1 is also increased in the serum of septic mice (9), while inhibition of TREM-1 protects mice from endotoxemia and cecal ligation and puncture-induced sepsis (8). Furthermore, an association between TREM-1 expression and disease activity has also been observed in non-infectious diseases such as autoimmune diseases (10, 11). In the samples of rheumatoid arthritis synovium, the expression of TREM-1 is upregulated, and increased numbers of TREM-1-positive cells are observed (10). In patients with inflammatory bowel diseases, TREM-1 expression in the intestine is increased and associated with disease severity (11). These studies suggest that TREM-1 can be a potential therapeutic target for immune dysregulation. However, the role of TREM-1 in radiation injury remains unknown. Considering the contribution of TREM-1 induction to dysregulation of the immune system, identifying the role of TREM-1 in radiation injury is of potentially scientifical importance.

Cold-inducible RNA-binding protein (CIRP) is an 18-kDa RNA chaperone expressed in various cell-types, including macrophages and neutrophils (12). Extracellular CIRP (eCIRP) levels in the circulation are increased in hypoxia, sepsis, and hemorrhagic shock and is identified as a damage-associated molecular pattern (DAMP) (12). eCIRP is released passively by cellular necrosis (12) or actively by the lysosomal exocytosis (13), exosomes (14), and Gasdermin D pore (15). Importantly, eCIRP has been shown to bind to TREM-1 and upregulate TREM-1 expression in macrophages (16). Despite the previous studies showing the implication of eCIRP in multiple disorders, whether eCIRP contributes to the pathogenesis of radiation injury has yet to be reported. In this study, we aimed to investigate the effect of radiation exposure on macrophage TREM-1 expression and the impact of TREM-1 on survival in radiation-exposed mice. Here we showed that ionizing radiation upregulated TREM-1 expression on macrophages *via* the increased release of eCIRP. Furthermore, knockout of TREM-1 improved survival after radiation exposure in mice. Thus, our findings suggest TREM-1 is a potential countermeasure target for radiation injury.

## Materials and methods

### Experimental animals and total body irradiation

Male C57BL/6 wild-type (WT) mice (8-12 weeks) were obtained from The Jackson Laboratory (Bar Harbor, ME). CIRP^−/−^ mice on the C57BL/6 background were originally obtained from Prof. Jun Fujita (Kyoto University, Kyoto, Japan). TREM-1^–/–^ mice, Trem1^tm1(KOMP)Vlcg^, were generated by the trans-NIH Knockout Mouse Project (KOMP) and obtained from the KOMP repository, University of California, Davis (16). Mice were housed in a temperature-controlled room on 12-hour (h) light/dark cycle and provided food and water. All experiments were performed following the guidelines for using experimental animals by the National Institutes of Health (Bethesda, MD). The animal protocol was approved by the Institutional Animal Care and Use Committees of the Feinstein Institutes for Medical Research.

Mice were exposed to 10-Gray (Gy) radiation using the Precision X-Ray X-RAD320 (Precision X-Ray Inc., North Branford, CT). Mice were gently restrained and placed in a transparent fitted container without any shielding during irradiation. The irradiation field was set to include the entire mouse TBI. In our *in vivo* experiments, peritoneal lavage was collected 24 h after radiation exposure, the cells were obtained by centrifugation at 300 × g and 4°C for 10 minutes to assess TREM-1 expression on peritoneal macrophages, and the supernatants were harvested to assess eCIRP levels in peritoneal lavage.

### Cell culture, isolation of primary mouse peritoneal macrophages, and radiation exposure

Mouse macrophage RAW264.7 cells were obtained from American Type Culture Collection (Manassas, VA) and cultured in Dulbecco’s modified Eagle’s medium supplemented with 10% heat inactivated FBS, 2 mM glutamine, and 1% penicillin-streptomycin at 37°C in 5% CO_2_. Primary mouse peritoneal macrophages were isolated from adult male C57Bl/6J mice (16). Briefly, mice were euthanized using CO_2_ asphyxiation and peritoneal cells were collected by washing peritoneal cavity with cold PBS and 2% FBS. The peritoneal cells were centrifuged at 300 × g and 4°C for 10 minutes and subsequently cultured in RPMI 1640 medium (Gibco, Grand Island, NY) with 10% heat inactivated FBS, 2 mM glutamine, and 1% penicillin-streptomycin. The number of cells were counted and plated in the cell culture plate. After 4 h nonadherent cells were removed and adherent cells, primary macrophages, were incubated in fresh RPMI 1640 medium at 37°C in 5% CO_2_ for subsequent studies. RAW264.7 cells and primary macrophages were exposed to radiation at different grays for different time periods using the X-ray Precision X-RAD320 (Precision X-Ray Inc.).

### Flow cytometry

RAW264.7 cells and peritoneal macrophages were collected at different time points (12 and 24 h after radiation exposure) and stained with BV421 anti-mouse TREM-1 antibody (BD Biosciences, Franklin Lakes, NJ). Data of TREM-1 expression were obtained by a BD LSRFortessa flow cytometer (BD Biosciences, San Jose, CA) with acquisition of 10,000 events and analyzed with FlowJo software (Tree Star, Ashland, OR). The frequency and mean fluorescence intensity (MFI) were used to evaluate the levels of TREM-1 expression. Peritoneal macrophages were recognized by F4/80 positive cells by staining with APC anti-mouse F4/80 antibody (BioLgend, San Diego, CA) for *in vitro* and *in vivo* experiments.

### Western blotting

Primary mouse peritoneal macrophages cultured in Opti-MEM (Thermo Fisher Scientific, Waltham, MA) were irradiated and culture supernatants were collected 24 h after radiation exposure. Peritoneal lavage was collected as previously described. Equal volumes of the cell culture supernatants and the peritoneal lavage were supplemented with 4× SDS sample buffer and run on NuPAGE 4–12% Bis-Tris gels (Invitrogen, Waltham, MA). The gels were transferred to nitrocellulose membranes (Invitrogen). After blocking with 0.1% casein in Tris-buffered saline for 1 h at room temperature, the membranes were incubated with the primary antibody of CIRP (Cat. No. 10209-2-AP; 1:1000, Proteintech, Rosemont, IL) overnight at 4°C. The membranes were washed with Tris-buffered saline and incubated with infrared dye-labeled secondary antibody for 1 h. The target bands were measured by an Odyssey imaging system (Li-Cor Biosciences, Lincoln, NE) and quantified using Image Studio software (Li-Cor Biosciences). Membranes were subsequently soaked in Ponceau S staining for 5-10 minutes to assess total protein levels.

### Quantitative reverse transcription-polymerase chain reaction

Total RNA was extracted by using the Illustra RNAspin Mini RNA Isolation Kit (GE Healthcare, Chicago, IL) according to the manufacturer’s instructions. One µg of total RNA extracted from cells was reverse transcribed into complementary deoxyribonucleic acid with reverse transcriptase (Applied Biosystems, Foster City, CA). The Polymerase chain reactions were performed in 20 μL of final volume containing forward and reverse primers (final concentration 0.06 μM each), cDNA, and SYBR Green master mix (Applied Biosystems, Foster City, CA) using a Step One Plus real-time polymerase chain reaction machine (Applied Biosystems). Mouse glyceraldehyde 3-phosphate dehydrogenase (GAPDH) mRNA was used as an internal control for normalization. Relative gene expression levels were calculated using 2-ΔΔCt method. Relative expression of mRNA was represented as fold change in comparison with vehicle group. The sequence of primers for this study is listed as follows: TREM-1, 5’-CTACAA CCCGATCCCTACCC-3’ (forward), and 5’-AAACCAGGCTCTTGCTGAGA-3’ (reverse); GAPDH, 5’-CATCACTGCCACCCAGAAGACTG-3’ (forward), and 3’-ATGCCAGTGAGCTTCCCGTTCAG-3’ (reverse).

### Survival study

To assess survival, C57BL/6 WT, CIRP^-/-^, and TREM-1^-/-^ mice were exposed to 6.5-Gy radiation using the X-ray Precision X-RAD320 (Precision X-Ray Inc.) and monitored the mice twice a day after radiation exposure for 30 days.

### Statistical analysis

All data are expressed as the mean and SEM. An unpaired two-tailed Student’s t-test was used for 2-group comparisons. One-way analysis of variance (ANOVA) and *post hoc* Tukey’s multiple comparison test were used for multiple groups comparison. Survival was analyzed by the Kaplan–Meier method and compared among the groups using the log-rank test. A two-sided *p* value of less than 0.05 was considered statistical significance. Data were analyzed with the use of GraphPad Prism graphing and statistical software (GraphPad Software, San Diego, CA).

## Results

### Radiation upregulates TREM-1 expression in murine macrophage cell line

**Figure 1:**
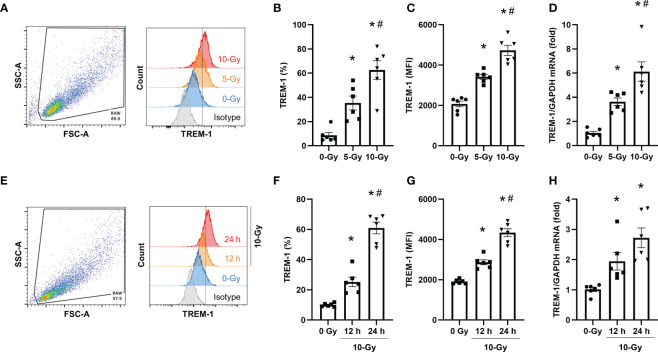
Radiation induces TREM-1 upregulation in macrophage cell line. **(A)** RAW264.7 cells were exposed to 5- and 10-Gy radiation and TREM-1 expression was assessed by flow cytometry 24 h after radiation exposure. **(B, C)** The frequency and MFI were used to evaluate the levels of TREM-1 expression. **(D)** Gene expression of TREM-1 was assessed by RT-qPCR 24 h after 5- and 10-Gy radiation exposure. The experiments were performed twice. Data are expressed as the mean ± SEM (n=6/group). The groups were compared by One-way ANOVA and *post hoc* Tukey’s multiple comparison test (*p<0.05 *vs*. 0-Gy, ^#^p<0.05 *vs*. 5-Gy). **(E)** RAW264.7 cells were exposed to 10-Gy radiation and TREM-1 expression was assessed by flow cytometry 12 and 24 h after radiation exposure. **(F, G)** The frequency and MFI were used to evaluate the levels of TREM-1 expression. **(H)** Gene expression of TREM-1 was assessed by RT-qPCR 12 h after 10-Gy radiation exposure. The experiments were performed twice. Data are expressed as the mean ± SEM (n=6/group). The groups were compared by One-way ANOVA and *post hoc* Tukey’s multiple comparison test (*p<0.05 *vs*. 0-Gy, ^#^p<0.05 *vs*. 10-Gy 12 h).

To determine the effect of radiation on TREM-1 expression, we first exposed RAW264.7 cells, a mouse macrophage cell line, to 5- and 10-Gy radiation and assessed the surface protein expression of TREM-1 at 24 h after radiation exposure by flow cytometry. We found that the surface TREM-1 expression was significantly increased after radiation exposure in a dose-dependent manner (**Figures 1A–C**). To determine whether radiation induces the *de novo* synthesis of TREM-1 in macrophages, we assessed TREM-1 gene expression in RAW264.7 cells by RT-qPCR. Consistent with the surface protein expression, we found that mRNA levels of TREM-1 were significantly increased by radiation exposure dose-dependently (**Figure 1D**). We also assessed TREM-1 expression on RAW264.7 cells after 10-Gy radiation exposure at different time points (12 and 24 h). We observed that 10-Gy irradiation significantly increased TREM-1 expression in a time-dependent manner (**Figures 1E–G**). TREM-1 gene expression after radiation exposure was also significantly upregulated time-dependently (**Figure 1H**). Taken together, radiation increases the mRNA and surface protein levels of TREM-1 in RAW264.7 cells dose- and time-dependent manner.

### Radiation upregulates TREM-1 expression in mouse peritoneal macrophages *in vitro* and *in vivo*

**Figure 2:**
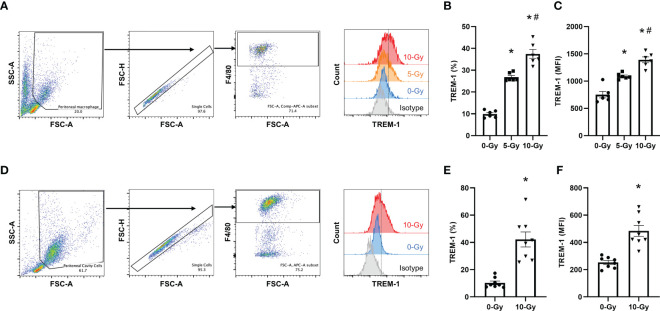
Radiation increases TREM-1 expression in peritoneal macrophages *in vitro* and *in vivo*. **(A)** Peritoneal macrophages were exposed to 5- and 10-Gy radiation and TREM-1 expression was assessed by flow cytometry 24 h after radiation exposure. **(B, C)** The frequency and MFI were used to evaluate the levels of TREM-1 expression. The experiment was performed twice. Data are expressed as the mean ± SEM (n=6/group). The groups were compared by One-way ANOVA and *post hoc* Tukey’s multiple comparison test (*p<0.05 *vs*. 0-Gy, ^#^p<0.05 *vs*. 5-Gy). **(D)** Healthy adult mice were exposed to 10-Gy radiation. Then, peritoneal cells were collected 24 h after radiation exposure and TREM-1 expression on peritoneal macrophages was assessed by flow cytometry. **(E, F)** The frequency and MFI were used to evaluate the levels of TREM-1 expression. The experiment was performed three times. Data are expressed as the mean ± SEM (n=8/group). The groups were compared by unpaired two-tailed Student’s t test (*p<0.05 *vs*. 0-Gy).

To further confirm that radiation upregulates TREM-1 expression in primary mouse macrophages, we next assessed it in primary macrophages *in vitro* as well as in peritoneal resident macrophages of irradiated mice *in vivo*. We isolated primary macrophages from the peritoneal cavity of healthy mice, exposed them to radiation, and then evaluated TREM-1 expression on peritoneal macrophages by flow cytometry 24 h after radiation exposure *in vitro*. Our results showed that 5- and 10-Gy radiation exposure dose-dependently increased TREM-1 expression on peritoneal macrophages compared to non-irradiation (**Figures 2A–C**). Based on these results, the radiation dose for the following *in vivo* experiment to evaluate the effect of radiation on TREM-1 expression was determined to be 10-Gy. We performed TBI of 10-Gy to healthy mice, collected peritoneal cells 24 h after radiation exposure, and assessed TREM-1 expression by flow cytometry. TREM-1 expression after 10-Gy radiation exposure was significantly increased compared to that without radiation exposure (**Figures 2D–F**). Thus, upregulation of TREM-1 expression after radiation exposure was verified in primary mouse macrophages.

### eCIRP release is increased and contributes to mortality in radiation injury

**Figure 3:**
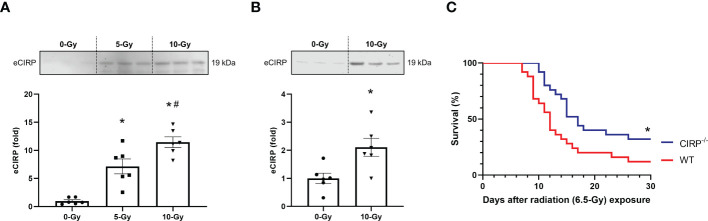
eCIRP release is elevated to contribute increased mortality in radiation injury. **(A)** Peritoneal macrophages were exposed to 5- and 10-Gy radiation. The supernatants were collected 24 h after radiation exposure and eCIRP levels were assessed by Western blotting. The experiment was performed twice. Data are expressed as the mean ± SEM (n=6/group). The groups were compared by One-way ANOVA and *post hoc* Tukey’s multiple comparison test (*p<0.05 *vs*. 0-Gy, ^#^p<0.05 *vs*. 5-Gy). **(B)** Healthy adult mice were exposed to 10-Gy radiation. Then, peritoneal lavage was collected 24 h after radiation exposure and eCIRP levels were assessed by Western blotting. The experiment was performed twice. Data are expressed as the mean ± SEM (n=6/group). The groups were compared by unpaired two-tailed Student’s t test (*p<0.05 *vs*. 0-Gy). **(C)** WT and CIRP^-/-^ mice were exposed to 6.5-Gy radiation and monitored for 30 days (n=25/group). Survival was compared among the groups using the log-rank test (*p<0.05 *vs*. WT).

We then sought to investigate the mechanism responsible for radiation-induced TREM-1 upregulation. We have previously reported that cell stress causes eCIRP release from macrophages and treatment of macrophages with recombinant murine CIRP (rmCIRP) directly upregulates TREM-1 expression (16). Thus, we hypothesized that radiation promotes the release of eCIRP, which then upregulates TREM-1 expression in macrophages. To demonstrate this hypothesis, we first assessed whether radiation causes eCIRP release. We exposed peritoneal macrophages to 5- and 10-Gy radiation and assessed eCIRP release in the supernatants of the cell-culture medium by Western blotting. We found that eCIRP levels in the culture supernatants after radiation exposure were significantly increased in a dose-dependent manner than those without radiation exposure (**Figure 3A**; **Supplementary Figure 1A**). Next, we exposed healthy WT mice to 10-Gy radiation and evaluated eCIRP levels in peritoneal lavage 24 h after radiation exposure *in vivo* by Western blotting. Our results showed that 10-Gy radiation exposure markedly increased eCIRP levels in peritoneal lavage compared to no irradiation (**Figure 3B**; **Supplementary Figure 1B**). We then performed a survival study in WT and CIRP^-/-^ mice exposed to radiation to evaluate the importance of eCIRP in radiation injury. Since 10-Gy radiation is mostly lethal for a survival study, the radiation dose of 6.5-Gy was chosen based on the previous studies on survival in radiation injury (4). We found that knockout in CIRP significantly improved survival in irradiated mice (**Figure 3C**). These findings indicate that eCIRP release is increased by radiation *in vitro* and *in vivo* as well as contributing to the survival in radiation injury.

### Knockout of CIRP attenuated radiation-induced increase in TREM-1 expression by macrophages

**Figure 4:**
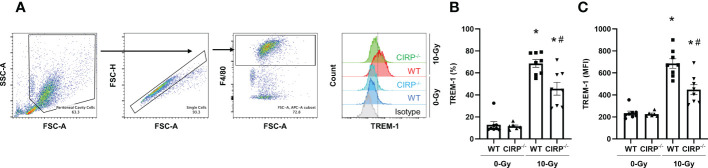
Knockout of CIRP attenuated radiation-induced increase in TREM-1 expression by macrophages. **(A)** WT mice and CIRP^-/-^ were exposed to 10-Gy radiation. Peritoneal cells were collected 24 h after radiation exposure and TREM-1 expression on peritoneal macrophages were evaluated by flow cytometry. **(B, C)** The frequency and MFI were used to evaluate the levels of TREM-1 expression. The experiment was performed three times. Data are expressed as the mean ± SEM (n=6-8/group). The groups were compared by One-way ANOVA and *post hoc* Tukey’s multiple comparison test (*p<0.05 *vs*. WT 0-Gy, ^#^p<0.05 *vs*. WT 10-Gy).

To demonstrate eCIRP induces TREM-1 upregulation during radiation injury, we exposed WT and CIRP^-/-^ mice to 10-Gy radiation, collected peritoneal cells 24 h after radiation exposure, and assessed TREM-1 expression on peritoneal macrophages by flow cytometry. The baseline expression of TREM-1 in macrophages was identical between WT and CIRP^-/-^ mice (**Figures 4A–C**). We found that 10-Gy radiation exposure significantly upregulated TREM-1 expression in WT mice compared to that without radiation exposure (**Figures 4A–C**), and the knockout of CIRP attenuated radiation-induced increase in TREM-1 expression by macrophages (**Figures 4A–C**). These findings indicate eCIRP contributes to TREM-1 upregulation during radiation injury. Together with the earlier data showing that radiation increases eCIRP release, we have demonstrated that TREM-1 expression on macrophages is increased by eCIRP, which is released from stressed cells during radiation.

### TREM-1 deficiency improves survival after radiation injury

**Figure 5:**
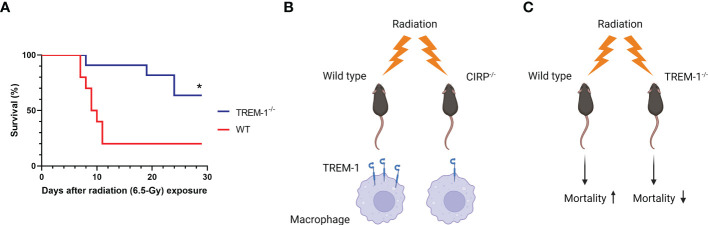
TREM-1 deficiency improves survival in radiation injury. **(A)** WT and TREM-1^-/-^ mice were exposed to 6.5-Gy radiation and monitored for 30 days (n=10 for WT mice and n=11 for TREM-1^-/-^ mice in each group). Survival was compared among the groups using the log-rank test (*p<0.05 *vs*. WT). **(B)** Ionizing radiation promotes eCIRP release from stressed cells and increases the mRNA and surface protein levels of TREM-1 in macrophages *via* eCIRP. **(C)** The TREM-1 deficient mice significantly improve 30-day survival after TBI.

Finally, to determine the impact of TREM-1 in radiation injury, we evaluated the potential benefit of TREM-1^-/-^ mice after TBI by comparing the survival between WT and TREM-1^-/-^ mice. We found that survival in TREM-1^-/-^ mice was significantly increased to 64% compared to 20% in WT mice after 30 days of 6.5-Gy TBI (**Figure 5A**). Thus, TREM-1 plays a critical role in mortality in radiation injury.

## Discussion

Immune dysfunction after radiation exposure and subsequent severe infections are critical prognostic factors in radiation injury (3, 4). Macrophages, which are radioresistant compared to neutrophils, potentially play a crucial role as innate immune cells for infections after radiation injury (5). TREM-1, a receptor expressed in myeloid cells, including macrophages, is upregulated in inflammatory diseases such as sepsis and its upregulation dysregulates the innate immune system, resulting in tissue injuries and poor prognosis (6). Therefore, we focused on TREM-1 expression on macrophages after radiation exposure and investigated the association between TREM-1 and prognosis in radiation injury. Our findings have shown that ionizing radiation upregulates mRNA and surface protein levels of TREM-1 in a mouse macrophage cell line. We have also demonstrated the upregulation of TREM-1 after radiation exposure in primary mouse macrophages *in vitro* and *in vivo* conditions. Furthermore, we have shown that radiation-induced TREM-1 upregulation is caused by eCIRP released from irradiated macrophages and that the TREM-1 deficient mice remarkably improve 30-day survival after TBI (**Figures 5B, C**).

Upregulation of TREM-1 expression on monocytes and neutrophils by lipopolysaccharide (LPS), bacteria, and fungi has been demonstrated *in vitro* (17). Furthermore, increased expression of TREM-1 has been observed in sterile conditions such as autoimmune diseases (10, 11), ischemia-reperfusion injury (18), myocardial infarction (19), and acute kidney injury (20). In these studies, the mechanisms of increased TREM-1 expression remained unclear. However, our previous study has shown that eCIRP directly upregulates TREM-1 expression in macrophages, shedding light on the mechanism of TREM-1 upregulation (16). Consistent with our previous study, here we have demonstrated a significant decrease in TREM-1 expression in CIRP^-/-^ mice compared to WT mice after radiation, supporting that eCIRP contributes to TREM-1 upregulation in different pathological conditions. The intracellular signaling pathways responsible for eCIRP-induced TREM-1 upregulation are not yet fully understood, but the downstream pathways of eCIRP’s receptors could be the potential candidates. Stimulation of TLR4, one of the receptors for eCIRP, causes NF-κB and MAP kinase activation, which could result in increased TREM-1 expression (12). Additionally, TREM-1 itself is also a receptor of eCIRP, and the phosphorylation of DAP12 and Syk, adaptor proteins of TREM-1, could also be the intracellular mechanism of TREM-1 upregulation (16). Furthermore, the eCIRP-TREM-1 axis may form a positive feedback loop that exponentially upregulates TREM-1 expression, which warrants further investigation in future studies. Since TREM-1 expression of irradiated CIRP^-/-^ mice was not reduced to that of sham mice, the effect of eCIRP on TREM-1 upregulation appears to be partial, suggesting the potential presence of other factors to enhance TREM-1 expression. Even though further studies are needed to bridge this gap, eCIRP can be regarded as one of the most important inducers of TREM-1 considering the degree of the reduction of TREM-1 expression in CIRP^-/-^ mice compared to WT mice after radiation. Furthermore, the improvement in survival was more significant in TREM-1 knockout mice than in CIRP knockout mice. This indicates that TREM-1 plays a more direct role in radiation injury compared to eCIRP, which serves as one of the major inducers for TREM-1 upregulation.

eCIRP has been known to be released from stressed cells due to hypoxia and LPS stimulation (12). In addition, multiple studies have focused on the mechanisms of eCIRP release in sepsis (13–15). On the other hand, it has not been studied whether radiation causes eCIRP release. Indeed, a recent study demonstrated that upon ultra-violet (UV) radiation, the expression level of CIRP increases; and it translocates to the cytoplasm from the nucleus in the cells (21). However, their study did not demonstrate whether these UV-irradiated cells released CIRP. In our present study, we have shown that eCIRP levels in the cell culture supernatant of peritoneal macrophages after radiation exposure and in peritoneal lavage isolated from irradiated mice were elevated, indicating radiation as a new stimulus to induce the release of eCIRP. The precise mechanism of eCIRP release by radiation remains elusive. DAMPs, in general, are regarded to be released as a cellular stress response to radiation exposure (22). Ionizing radiation causes double-strand DNA breaks, ideally the cells should either resolve the damage through repair mechanisms or undergo regulatory cell death, including apoptosis or cellular senescence, to limit the release of cellular molecules (23). The concept of immunogenic cell death induction after radiation exposure has been reported, which includes necroptosis and ferroptosis (22). These cell deaths are known to be accompanied by the release of DAMPs in large amounts because of an uncontrolled membrane rupture (22). Therefore, it is plausible that eCIRP, one of the DAMPs, can be released due to certain kinds of immunogenic cell deaths after radiation exposure.

Our data have shown that genetic depletion of TREM-1 in mice improves 30-day survival after irradiation. Knockdown of TREM-1 has been reported to downregulate inflammatory chemokines and cytokines such as MCP-1, CXCL10, IL-1β, as well as IL-18 in response to LPS *in vitro* (24, 25). It has also been shown that inhibition of IL-18 ameliorates multiple organ damage and significantly increases 30-day survival in mice after 9-Gy TBI (26). Thus, TREM-1 might exacerbate organ injury *via* the production of inflammatory mediators to worsen outcomes in radiation injury. Another possible mechanism of increased mortality by TREM-1 in radiation can be TREM-1-mediated pyroptosis based on the following studies. Knockout of NOD-like receptor family pyrin domain-containing 3 (NLRP3) reduces pyroptosis in bone marrow-derived macrophages following irradiation *in vitro* and NLRP3 knockout mice show improved 30-day survival after 9.5-Gy TBI *in vivo* (27). A study has also demonstrated that TREM-1 induces NLRP3-mediated pyroptosis (28). Additional studies are awaited to confirm the concept of TREM-1-mediated pyroptosis in radiation injury. Infection is the major factor which directly causes death after radiation (3, 4). We have recently shown that eCIRP suppresses macrophage bacterial phagocytosis by STAT3 phosphorylation followed by the formation of STAT3-βPIX complex, which leads to Rac-1 inhibition (29). Even though the role of TREM-1 was not assessed in that study, upregulation of TREM-1 by eCIRP after radiation exposure may impair the phagocytic function of macrophages, considering the involvement of the eCIRP-TREM-1 axis reported in different settings. Nevertheless, whether TREM-1 affects phagocytic dysfunction during radiation injury remains unknown and thus may require future studies.

Therapeutic intervention targeting TREM-1 using pharmacological inhibitors, such as LP17, M3, and LR12, would also be interesting. The peptide LP17 consist of a highly conserved region in the extracellular portion of TREM-1 and acts as decoy receptor of TREM-1 (9). The peptide M3 has been developed as a novel peptide designed to inhibit the interaction between eCIRP and TREM-1 (16). Pharmacological suppression of TREM-1 with either LP17 or M3 attenuates organ injury markers organ injury markers in serum and the lung injury score and improves survival in septic mice (16). In sterile conditions, such as hepatic ischemia-reperfusion injury, M3 has been shown to reduce tissue injury and improve prognosis (18). The peptide LR12 is composed of 12 amino acids with anti-inflammatory effects in the LP17 sequence, which also acts as decoy receptors of TREM-1 and protects against sepsis-induced cardiovascular dysfunction and multiple organ failure (6, 30). Furthermore, LR12 has already been used under Phase 2 clinical trials in certain diseases including sepsis (6, 31). To date, the efficacy of TREM-1 inhibitors on radiation injury has not been evaluated. Given the significance of the survival improvement of TREM-1 knockout mice in the present study, TREM-1 inhibitors have the potential to become novel therapeutic drugs against radiation injury.

We used macrophages in our *in vitro* settings and assessed TREM-1 levels of peritoneal macrophages *in vivo* since TREM-1, as its name suggests, is mainly expressed on myeloid cells, such as macrophages. However, neutrophils as well as other immune and non-immune cells also express TREM-1 to some extent. Inhibition of TREM-1 expressed on renal endothelial cells reduces the production of inflammatory cytokines and angiogenic factors after stimulation with DAMPs, potentially attenuating acute kidney injury (20). Furthermore, knockdown of TREM-1 on cardiomyocytes attenuates cardiomyocytes pyroptosis after LPS stimulation (25). Thus, it is possible that not only TREM-1 on macrophages but also that on other cell types, such as neutrophils and endothelial cells, could have contributed to our survival data of TREM-1 knockout mice. Nevertheless, the main aim of this study is to address the importance of TREM-1 in radiation rather than limiting it to macrophages. Further studies on the status of TREM-1 in other cell types besides macrophages would be of importance in the future. Moreover, we have shown that TREM-1 expression is decreased after irradiation in CIRP knockout mice but have not investigated the survival of CIRP knockout in this study. However, considering that high dose radiation can cause sepsis due to immune dysfunction (3) and CIRP knockout mice exhibit attenuated organ injuries and a marked increase in survival after sepsis (12), CIRP knockout has the potential to improve the prognosis by improving sepsis after irradiation, which might be a future research interest.

Throughout this paper, we used a radiation dose of 10-Gy for the short-term *in vivo* experiments. TREM-1 expression in peritoneal macrophages and eCIRP levels in the peritoneal fluid of mice were examined after 10-Gy radiation exposure, as this dose induces significant immune responses in mice (32). However, previous publications have suggested that 10-Gy is too lethal to perform a survival study as mentioned earlier. As such, we performed survival studies at a lower dose of radiation, i.e., 6.5-Gy, which did not depict 100% lethality at 30 days. In addition, we found that 5- and 10-Gy radiation significantly increased macrophage TREM-1 expression and eCIRP release *in vitro*, as shown in **Figure 3A**. Given that 6.5-Gy is between the 5- and 10-Gy doses, it is reasonable to assume that TREM-1 and eCIRP levels could also be increased at this radiation dose.

In summary, ionizing radiation increases TREM-1 expression in macrophages *via* eCIRP release, and TREM-1 is an independent factor to worsen survival after total body irradiation. These results suggest a potential novel approach against radiation injury by targeting the eCIRP-TREM-1 axis.

## Data availability statement

The original contributions presented in the study are included in the article/**Supplementary Material**. Further inquiries can be directed to the corresponding author.

## Ethics statement

The animal protocol was approved by the Institutional Animal Care and Use Committees of the Feinstein Institutes for Medical Research.

## Author contributions

SY, AM, and MA designed experiments. SY, AM, and GM performed *in vitro* and *in vivo* experiments. SY and GM exposed cells and mice to radiation. SY, AM, MA, and MB analyzed the data. SY, AM, and MA wrote the manuscript. PW reviewed and edited the manuscript. PW conceived the idea and supervised the project. All authors contributed to the article and approved the submitted version.
