# Supplementary figures and images for: Radiation upregulates macrophage TREM-1 expression to exacerbate injury in mice
Source: Front Immunol. 2023 Apr 24;14:1151250. doi: 10.3389/fimmu.2023.1151250 (PMC10164953; doi:10.3389/fimmu.2023.1151250)

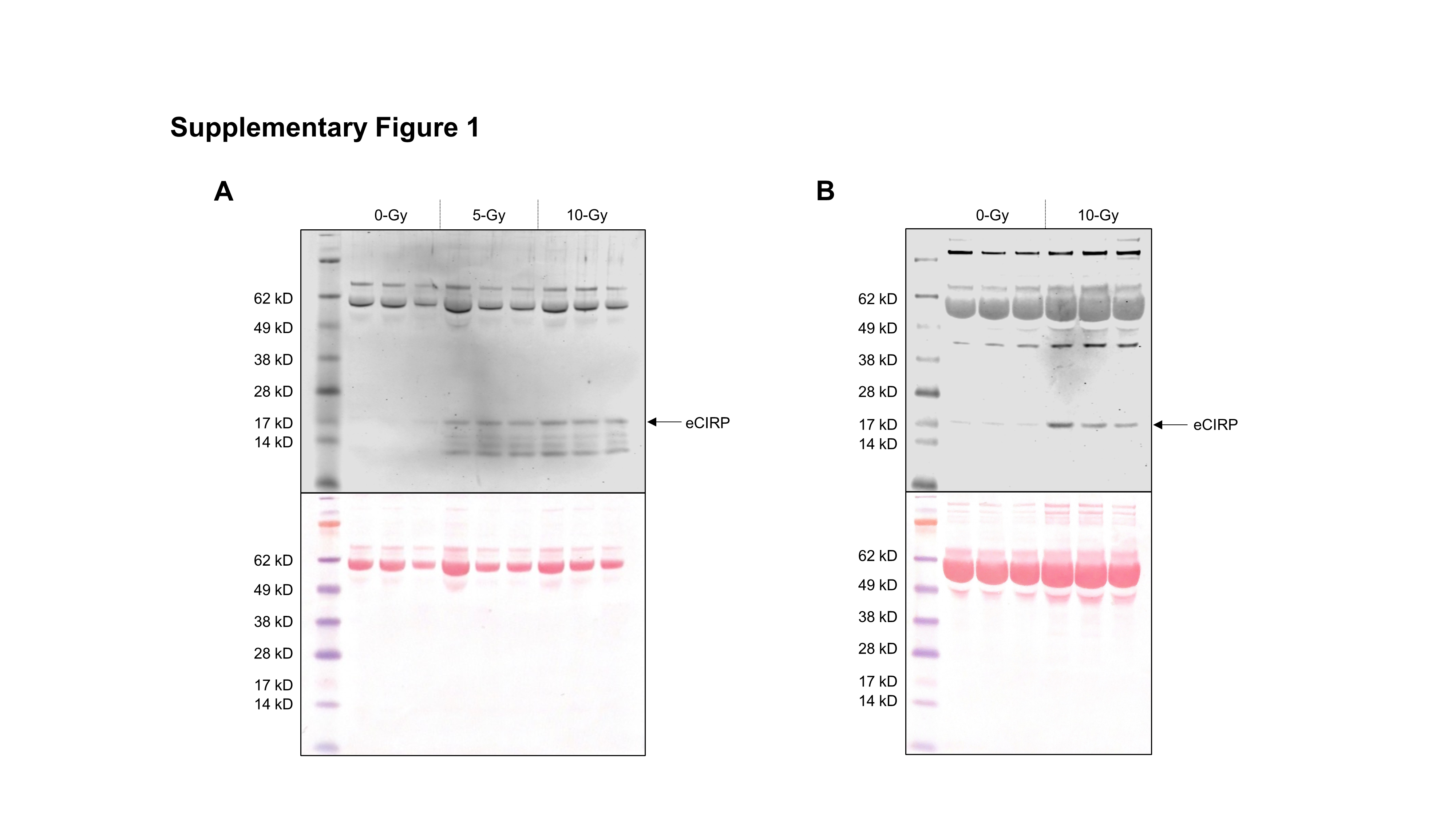

Supplement: Supplementary file 1 [file Image_1.tif]
